# Some Medico-Legal Aspects of Trauma in Relation to Diseased Cerebral Arteries1Fragment of president’s address before the American Microscopical Society, held at Columbus, O., August 17-19, 1899.

**Published:** 1899-10

**Authors:** William C. Krauss

**Affiliations:** Buffalo, N. Y.


### SOME MEDICO-LEGAL ASPECTS OF TRAUMA IN RELATION TO DISEASED CEREBRAL ARTERIES.[1]

By WILLIAM C. KRAUSS, M. D., Buffalo, N. Y.

ONE of the most interesting questions in neurology, and one of the most important is the relation of trauma to disease of the nervous system. I do not mean a trauma sufficient to produce a fracture if applied to the cranium, or even to a laceration, if applied to the soft tissues, but a trauma apparently slight and of seeming little import at the time of its infliction. To those cases especially do I refer where serious, even fatal results follow a slight contusion, in certain individuals, while in others the same force would be scarcely if at all perceptible; and this brings me to the important and valuable services to be rendered by the microscope in elucidating the true cause of the various phenomena noted.

1. Fragment of president's address before the American Microscopical Society, held at Columbus, O., August 17-19, 1899.

It is a well-known fact that the susceptibility of individuals differ; that is, what produces a serious condition of things in one would have no effect upon another. This susceptibility may be evidenced in one of two ways: first, by producing a class of disorders comprised under the head of the traumatic neuroses, usually functional and temporary, and, secondly, by calling forth a more serious class of disorders, which terminate in death itself, either primarily or through sequellæ. Such are injuries to the arterial system, weakened and degenerated by disease, either acquired or inherited. The vices, alcoholism and syphilis, produce upon the arterial walls, especially of the cerebral vessels, a degeneration and disintegration, which render them unsafe carriers of the blood even under the usual, normal blood pressure. When the pressure is increased, the tension becoming greater, then there develop, as Charcot pointed out in 1868, and Virchow previously in 1851, under the name of *ectasia ambulare*, small aneurismal protuberances, which under slight provocation, as shock, excitement or exertion, physical or psychical, burst, and permit the egress of blood under the high arterial tension into the surrounding brain tissues.

Of the two classes of disorders the first or functional are of less serious import and depend less upon a preëxisting pathological condition of the constituent elements of the nervous system than upon a general debasement or deterioration of the whole nervous organisation. There is found in those individuals an increased morbid reaction of the ganglionic nerve centers to all kinds of impressions, both mental and bodily, whether slight or profound, and is liable to manifest itself on very short notice and on the least provocation.

The microscope has been unable as yet to point out the degree of involvement of these nerve centers or even the area of involvement, but may at some future time render inestimable aid in solving one of the most complex questions in human neurology. This very absence of knowledge regarding its pathology has led to much controversy and debate regarding this affection. There are even some who look upon it with contempt under the mistaken idea that the barometric changes of disposition, tastes and feelings from day to day indicate a fraudulent basis. Studied originally by Erichsen, under the name of railway spine, this syndrome has been investigated by Rigler, Hodges, Page, Clevenger, Oppenheim, Strümpell, Dana, Outten, Knapp, Bailey, and in no uncertain way by Charcot and his pupils, and has received a galaxy of names, polyonymic as well as mononymic. The relation which the arterial system bears to the genesis of this affection is as yet not well defined, except in this much, that an artery diseased is a poor conduit for the passage of a fluid whose function in the brain is primarily to nourish and regenerate tired or worn out nerve cells.

Turning to the second class of disorders we meet a condition of the vessel walls long since recognised, and easily demonstrable by any one acquainted with microscopic technique. Doubt and uncertainty regarding the lesion, has long since disappeared and the student beginning the study of medicine is taught at the bedside and demonstrated in the laboratory the effects of an endarteritis and the structural appearance of the lesion. He is, moreover, taught that this condition of the vessel walls, leading to their deterioration, disintegration and destruction when occurring in early manhood is attributable, in the great majority of cases, to two vices—alcoholism and syphilis—both unfitting the arteries for the work nature originally mapped out for them.

Three conditions are necessary for the normal circulation of the blood in the arterial system. First, the arterial walls must be capable of dilating without strain or injury to any of the coats of the arteries; second, the walls must be able to contract upon their contents, and third the lumen of the vessels must be preserved.

The elasticity of the vessel walls permits the blood to flow in an unbroken continuous current through the smaller bloodvessels and arteries, relieving them of the direct force of the heart's action. In fact the main force of the heart is spent in distending the larger arteries and the immediate propelling force of the circulation is the elasticity of the arteries in which the heart stores up the energy of its systole for the moment. The blood pressure is, of course, highest in the heart, considerable in the whole arterial system, though gradually diminishing toward the capillaries, in which it would be feeble; lowei still in the smaller veins and at its minimum where the great veins enter the heart. It is estimated that the blood pressure of the carotids in man is not less than 150 to 200 millimeters of mercury. To supply a sufficient elasticity to the arterial walls, to withstand the force of the heart's action, the middle coat has been supplied with yellow elastic tissue, the importance of which, when diseased, is not to be overlooked or underestimated.

The contractility of the arteries has great physiological importance, but less pathological than the elasticity. In the smaller vessels by virtue of their contractile walls, the distribution of blood is regulated to the various organs. Where the resiliency of the vessels is at fault, active or passive anemias or hyperemias of the brain and other organs are produced.

When the elasticity and contractility of the vessels are not impeded by disease and the lumen is not encroached upon, nutrition is carried on normally and the functions of the parts supplied are commensurate with their growth and development. Where the vessel walls are weakened by disease, the elasticity of the arteries is impaired, the resistance to the blood pressure is too feeble and hemorrhage from rupture takes place.

The disease leading to the weakening of the vessel walls has been variously designated by different names as arterio-sclerosis, atheroma, chronic endarteritis, endarteritis deformans and the like.

A pure and simple physiological process of old age, its earlier occurrence or the more extreme grades of its severity are dependent upon some toxic principle in the organism, prominent among which may be mentioned, syphilis, chronic alcoholism, gout, uremic manifestations and in some cases it is due to overstrain. Although the causes of arterio-sclerosis are well known, there exists diversity of opinion as to how these recognised causes operate. The opinion once in vogue that the inflammation started in the inner coats as a result of the irritation of the toxic or infectious agencies in the blood is no longer tenable. It seems fairly well established that the degenerative changes and loss of elasticity in the vessel wall are the result of the primitive causes, and the hyperplastic processes in the intima and other parts of the arterial wall are the ultimate result. (Stengel.)

Thoma believes that the degenerate vessel tends to dilate and to thereby slow the circulation; the slowing leads to hyperemia of the vessels, and to a consequent connective tissue new formation in the intima, which narrows the vessels. These changes also occur in the middle and outer coats.

Heubner, in 1874, first described accurately the changes taking place in the arteries, especially at the base of the brain, resulting in an arteritis or endarteritis, and found existing either as an independent disorder or as part of a local syphilitic affection. An endarteritis is found, due to the pouring out of round cells from the *vasa vasorum*, an endothelial proliferation, followed by thickening of the intima, fenestrated membrane and adventitia. As a result, the lumen of the vessel is narrowed, even occluded, or the intima becomes roughened and changed and a thrombus forms, which may in turn give rise to an embolus.

The process may be a diffuse one, but it is most commonly distributed irregularly, the inner surface of the vessel exhibiting patches of sclerosis separated by areas of comparatively healthy tissues, or is furrowed or wrinkled with irregularities. In many cases also there occurs a deposition of new material within the affected area of the vessel wall, this newly formed tissue being in some cases hyaline and nearly structureless ("hyaline degeneration"); in others containing round cells, few or many, embedded in a hyaline or gelatinous matrix. This newly formed tissue, furthermore, is very prone to early and extensive, slowly progressive, degenerative changes, fatty or, less frequently, calcareous in nature, which render the diseased areas more distinctly visible, giving rise to the familiar "atheromatous plaque."

In those cases in which the degenerative change is calcareous in character, the formation of irregular chalky masses, or of smooth "calcareous plates," is a common result. In extreme cases these deposits may involve the entire circumference of the vessel for long distances, rendering the arterial wall so brittle that instead of bending it fractures under application of force. The fatty and calcareous degenerative changes are often combined; indeed, in the more serious cases this is the general rule, the deposition of calcareous matter appearing as a later stage of the fatty degenerative atheroma.

The patches of atheroma are raised, usually encroaching slightly upon the lumen of the artery, although in ordinary cases they interfere only in a very minor degree with blood-flow.

There are three alterations which atheroma produces, each of which may, according to circumstances have important effects on the circulation.

These are narrowing of the caliber, loss of elasticity and rigidity of the wall and interference with the muscular contractility of the vessel. Thus it is seen that this process counteracts each one of the conditions so necessary for the normal circulation of the blood. Generally as a result of the weakening of the vessel walls by sclerosis or degeneration small saccular aneurisms, often called miliary aneurisms, develop in the end arteries of the brain, but especially in the arteries supplying the optic thalmus, corpus striatus and lenticular body, and the lenticulo-striate artery has even been designated by Charcot the apoplectic artery, because of its proneness to aneurismal development and consequent rupture.

Hemorrhage rarely occurs at the cortex of the brain because the smaller arteries and arterioles enjoy an intimate anastomosis with each other and any sudden increase in blood pressure is quickly equalised through this anastomosis. Then, too, these arteries do not receive the direct flow of the blood from the carotids, and the blood pressure is perceptibly diminished by the time it reaches these arterioles. Arteries at the base of the brain are not so liable to rupture as the end arteries going to the large ganglia, because of their anastomoses in the circle of Willis and because of their tortuous course, which permits of considerable increased pressure before they straighten out and become tense. Aneurisms of small size, from a pea to a bean, are very prone to develop along these arteries and hence are next in importance to the lenticulo-striate artery as regards rupture.

The end arteries in the region of the internal capsule are so liable to rupture, because instead of having a system of anastomoses, they terminate in blind pockets.

When the large vessels at the base of the brain, that is, of the middle cerebral, posterior cerebral and the communicating arteries, have undergone calcareous degeneration the absence of the normal elasticity of these vessels allows the blood to pass with great force and directness into these end arteries and if the conditions are favorable, that is an endarteritis is present, the vessel walls will yield to the strain and become bulbous and then aneurismal. They may remain in this condition for some years even, but they are comparable to the faulty flues in a boiler—only safe as long as the pressure is low. When the pressure is increased physiologically, through eating, drinking, sexual congress, exercise, or pathologically, through stimulants, the emotions, or by slight injuries to the head, then the aneurismal walls give way and a serious hemorrhage follows.

Without previous disease of the arteries it is almost impossible to think of hemorrhage from rupture to take place. The action of the heart alone is never responsible. In most of these cases where the endarteritis has existed for a long period, the heart is hypertrophied, a condition probably due to the resistance which it has to overcome in forcing the blood along through the diseased and often narrowed vessels without the aid of the normal elasticity by which the work of the heart is much lessened.

To an individual with such a diseased condition of the cerebral arteries, a trauma, be it ever so slight, may, and does very often, produce fatal hemorrhages. When such cases arise in the criminal courts, what is to decide the true guilt of the prisoner? Should the jury listen only to the passioned eloquent summary of the prosecuting attorney, or should the jury take cognizance of the status of the cerebral vessels of the victim previous to the injury, and heed the testimony of the microscopist, who has carefully examined into the condition of the arteries and found the prisoner, though guilty, but nevertheless, a victim of circumstances?

There is an old story told of a physician who when being asked his age, replied that he was as old as his arteries. How true this is, those of us who feel of arteries can bear testimony, but is it not equally true, that a man is no healthier than his arteries? Here again, those of us who examine applicants for life insurance know full well that no individual with a sclerotic radial or temporal artery will be accepted as a first class physical risk. If a condition of sclerosis exists at the wrist or forehead, it is safe to infer that the same condition will be found at the base of the brain. It has even been asserted by Duret that endarteritis will be found affecting the basilar artery when its presence can be detected in no other artery of the body.

If life insurance companies regard such individuals as substandard risks, why should not courts of justice? I do not make this plea because I favor the criminal classes or desire to lighten their punishment in any degree, or to offer any inducement or incentive to future transgressors, but simply to call attention to a question which I believe will in the near future be given full and due consideration.

A chain is no stronger than its weakest link, or a bridge stronger than its weakest span, or a man stronger than his weakest artery.

If a man with such a condition of the arteries be engaged in heated discussion, in mental or physical excitement, or is under the influence of alcoholic drinks, or all combined, and is suddenly stricken down with a fatal hemorrhage, the verdict will simply read, death from apoplexy. If, however, he is engaged in a dispute, under the same conditions as just noted, and receives a slight blow, perhaps even a push by his antagonist, and a fatal hemorrhage likewise ensues, the verdict will read, murder or manslaughter and the prisoner will most generally receive the full punishment allotted by the statute. A case of this kind, in which I was engaged as medical expert, but failed for some reason to appear on the stand, illustrates the injustice of such procedures, and although perhaps not admissible in an address of this kind, nevertheless, I will report it and grant you the exceptions.

The victim was a sailor of Swedish extraction, and had sailed the lakes for seven or eight years, making his home at Buffalo. He was in the habit of spending his nights when on shore at a notorious dance hall in the infected district. One night he met a singer in the resort, whose husband was "the strong man," doing certain tricks, as stone-breaking, tearing chains asunder and the like. The couple proceeded up-stairs to a private room and drank heavily of strong liquors. Leaving the room and descending the stairs they met the husband who struck the sailor on the jaw, felling him, and in a few moments the latter expired. Post mortem examination of the cranial cavity revealed a large flattened clot of blood in the posterior fossa of the cranium—the same having escaped from a large opening in the basilar artery, near its bifurcation into the posterior cerebrals. The basilar artery was the seat of an arteritis, also endarteritis, with thickening of the lumen in some places and thinning in others. It was at one of the thinned portions where the rupture occurred. Other evidences of cerebral or arterial disease were wanting, and the anatomical diagnosis was reported as "cerebral basilar hemorrhage."

Of course, no history of syphilis could be obtained, but the seat of disease, condition of the artery, occupation, habits and surroundings of the man, leave little doubt as to some previous specific inoculation. No doubt the woman's husband was, to a great degree, a victim of circumstances. With the degenerated condition of the arteries, as was found in the case, the excitement might of itself have produced the arterial rupture, and this, further accentuated by the increased arterial tension, due to the large amount of stimulants which he had taken, would have required but a very slight shock, either mental or physical, to have produced a disruption of the diseased vessel. The force of the blow was of itself not so important under these circumstances, although at the trial much stress was laid on the assumption that the prisoner must have dealt a terrible knock-out blow, being noted for his strength, which, in fact, consisted only in stage tricks, he being actually possessed of only ordinary strength.

There was no examination made microscopically to determine the exact pathological condition of the bloodvessels. Neither was the defense of the prisoner as vigorously fought as might have been on account of the evil atmosphere surrounding the tragedy, and the verdict of the jury read manslaughter in the first degree. Had the microscope been called into this case, the arterial degeneration probably present been thoroughly demonstrated to the court, the defense of the prisoner carried out on lines suggested above, I believe the disposition of the case would have been different, even though it was of unsavory character.

Soon after I was called into another medico-legal case, which, with your kind permission, I will also briefly narrate.

A policeman making his rounds in the lower part of our city came across a group of children surrounding a drunken man, who had fallen to the ground. In trying to rouse him, the man suddenly sprang to his feet and attacked the policeman, so that the latter was obliged to defend himself, and in the fray, struck the drunken man on the head with his club. The man staggered and fell to the ground. An ambulance was called and he was taken to an emergency hospital for treatment. No fracture of the skull was found, and no abrasion of the scalp could be detected, but the patient was in a condition of coma—very light—which increased gradually to a deep coma. A meningitis was supposed to be the cause of the coma and after an illness of two weeks he died. The autopsy, made very carefully, did not reveal any meningeal inflammation, but on careful inspection a small aneurism with hemorrhage into the medulla was detected. The brain and arteries were carefully hardened and prepared for microscopical examination, which was conducted by Dr. H. U. Williams, of Buffalo, representing the prosecution, and by myself for the defense. No trace of any injury to the meninges could be found. The brain matter was healthy; not so, however, the arteries. A general endarteritis was found affecting nearly all the cerebral arteries, but more especially the vessels at the base of the brain—the vertebrals and the basilar. The middle coat of the vessels were unequally thickened and thinned and a small aneurism had developed near the junction of the basilar and vertebral arteries. This aneurism was furthermore ruptured and the pressure of the escaped blood upon the vital centers in the medulla occasioned the man's death.

At the trial, the medico-legal question of most import was in how far did the blow of the policeman's club produce death? Had the arteries been in a normal healthy condition there would have been no contest over the case, no point of difference to be settled. The condition of the vessel walls, however, put another aspect to the case. Who could say but what the excitement and the consequent increased arterial pressure might not of itself have produced a rupture of the diseased vessels. The microscope proved conclusively a dangerous condition of the ruptured vessel and case after case has been found of a similar death without any external violence. It was therefore impossible to state what brought about the rupture and the jury wisely exonerated the policeman from the charge brought against him.

Diseased conditions of the cerebral arteries, especially produced by alcohol and syphilis—place individuals into a class peculiarly of their own. They are dangerous to themselves and far more dangerous to others. They offer a point of least resistance, and upon the degree of resistance does their own life and safety depend. They are no stronger, no healthier than their diseased vessels and should be so regarded in all courts of justice.

A trauma not severe enough to produce any pain or injury to a normal healthy man will produce brain lesions of the most dangerous character in these cases. Where the condition of the arteries is not made manifest to the court by a microscopist or where the court fails or refuses to see the susceptibility of the patient to apoplexy through slight injuries, then the prisoner receives punishment far in excess of what he really deserves. All cases of death following head injuries should be most carefully investigated and the true condition of affairs be made known in no uncertain manner. The microscopist becomes in all such cases a most important adjunct and his findings and deductions should be given most careful and respectful consideration.

Turning now to another class of symptoms engendered by trauma to the head, we meet disturbances of mental action, sometimes slight, sometimes profound and not in a few cases of beginning degenerative lesions, terminating in insanity. It is not so much the local effect of the injury, but the general effect of a *commotio cerebri*, and the syndrome of mental disorders induced by such cause has been well termed by the Germans "commotion insanity." The effect of a violent blow or jar, or jolt to the head must have some influence upon the molecules of the brain, as well as upon the encephalon as a mass: it must displace and disarrange delicate microscopic structures, such as the nerve cells and nerve fibers.

If there be present in the individual the remnant of previous syphilitic inoculation, the effect will be far reaching and most serious. A slumbering paresis is many times awakened by slight accident so infinitesimal that at the time no heed is paid to it. Slowly and surely the progressive symptoms of paresis develop and soon the disease is converted into a galloping paresis, and fortunately for the patient and friends a rapid dissolution is to be looked for. The microscope again discloses the cause, as Mickle, of London; Mendel, of Berlin, and many others have long since shown, a degenerative condition of the frontal lobes with a previous syphilitic inoculation as a background.

General paresis, like tabes, has an initial stage that may last for months or years during which the patient is not only not incapacitated for work, but may conduct himself so rationally that no suspicion is entertained that he is already suffering from a disease which is soon to destroy both body and mind. From the insidiousness of its onset it is usually impossible to say even approximately when the morbid process began. In nontraumatic cases, where the first marked symptoms consist of an attack of acute maniacal excitement, or of acute mental depression, there is every reason to suppose that the disease had already existed, though unsuspected, for sometime. Similarly, when an injury to the head is quickly followed by an outbreak of the symptoms of the disease, it is never possible to say with absolute certainty that the traumatism did anything more than hasten into activity a process which was already existent and whose ultimate development was inevitable, irrespective of traumatic agency. (Bailey.)

This early prodromal stage, when the individual is thought by friends and relatives to be perfectly sound and healthy, is called by LeGrand du Saulle the "medico-legal period," because of the large number of medico-legal inquiries made bearing upon the patient's soundness of body and mind during this period. I recall a case where in such a predisposed individual paretic symptoms rapidly developed after having been knocked down on a street corner by a careless driver. Previous to this accident he was a thorough musician and an adept violinist. After a few days he became a paretic of the most pronounced type with his delusions of grandeur all concentrated about music, opera and song. The family, on advice of the consulting attorney had recourse to law and a suit of damages was the result. Trauma was looked upon as the cause of this man's insanity, but as a matter of fact it was only contributory. The real cause was the pathological condition existing in the poor fellow's brain—a product of his own misdeeds. The trauma only lit up the slumbering embers and once flared-up the progress of the disease was not to be controlled.

The percentage of cases of paresis, due to trauma is given by Schlager[1] as "one-seventh of all cases of mental diseases induced by head injuries." Meyer found 15 cases of injury to the head in 76 cases of general paresis in which the causes were clearly made out. In 80 male cases Krafft-Ebing found cranial injury to be the cause in 6. Christian observed 43 cases of general paresis in 100 cases of injuries to the skull. Other observers as Mickle and Gudden, have found a relationship between trauma and general paresis in 7-10 per cent. of their cases.

1. Quoted from Bailey; Accident and injury.

In the great majority of these cases the head injury is but secondary to the predispositions underlying the individual. These may be either inherited or acquired. If the former, then a congenital syphilis; if the latter, a preceding syphilitic inoculation are, as a rule, the predisposing factors. Generally, and if the courts are ill-advised and ignorant of the underlying condition, punishment usually in the shape of heavy damages are awarded the patient.

The rôle which the diseased bloodvessels play in paresis is perhaps primary, leading to the atropic changes in the brain cortex itself. The very earliest anomalies are nutritive defects in the ganglionic cortical elements, and then follow local hyperemias of the frontal, temporal or parietal convolutions, dilatation and degeneration of the coats of vessels, lymphatic stasis and effusions into perivascular lymph spaces, swelling and then wasting of nerve cells and fibers, proliferation of neuroglia and of protoplasmic glia cells, cortical and meningeal adhesions and the formation of neo-membranes.

Thus it will be seen that although differing materially from the pathological condition of the arteries, as found in apoplectic attacks, nevertheless, the degenerative coats of the artery, through syphilitic infection, are equally as dangerous to the victim and as calamitous to the aggressor. . . . . . . . . . . . . . . . . . . . .

Alcohol and syphilis, the two vices of civilisation, produce the most degenerating conditions in the neuron and in the vessels going to support and tone the system of neurons. Death and disease follow closely upon their trail; they single out with great delight both youth and beauty; they render the strong and vigorous weak and susceptible; they disintegrate and destroy the physical, as well as the psychical; but more than all, not satisfied with their own unfortunate prey, they tend to inculpate others by force of circumstances and weave a web of guilt and suspicion about them. Fortunately, however, in many cases they leave such glaring earmarks that the microscope is able to detect their presence and act thereby as a safeguard to the unsuspecting and innocent.

371 Delaware Avenue.

---

The following, says the *Medical Record*, is more or less good advice for doctors: "Drink less, breathe more; eat less, chew more; ride less, walk more; clothe less, bathe more; worry less, work more; waste less, give more; write less, read more; preach less, practice more."

